# Retraction: Disposable screen-printed sensors for determination of Duloxetine Hydrochloride

**DOI:** 10.1186/1752-153X-6-72

**Published:** 2012-07-24

**Authors:** Nawal A Alarfaj, Reda A Ammar, Maha F El-Tohamy

**Affiliations:** 1 Department of Chemistry, College of Science, King Saud University, P.O. Box 22452, Riyadh, 11495, Saudi Arabia; 2 Department of Chemistry, Collage of Science, Al-Azhar University, Cairo, Egypt; 3 Collage of Science, Chemistry Department, KSU, Riyadh, Saudi Arabia

## Retraction

This manuscript [1] has been retracted for inappropriate and unattributed text duplication from a previous paper published in Drug Testing and Analysis [2].
